# Perceptions of Competence, Strength, and Age Influence Voters to Select Leaders with Lower-Pitched Voices

**DOI:** 10.1371/journal.pone.0133779

**Published:** 2015-08-07

**Authors:** Casey A. Klofstad, Rindy C. Anderson, Stephen Nowicki

**Affiliations:** 1 University of Miami, Department of Political Science, Coral Gables, FL, United States of America; 2 Duke University, Department of Biology, Durham, NC, United States of America; 3 Florida Atlantic University, Department of Biology, Boca Raton, FL, United States of America; UNLV, UNITED STATES

## Abstract

Voters prefer leaders with lower-pitched voices because they are perceived as stronger, having greater physical prowess, more competent, and having greater integrity. An alternative hypothesis that has yet to be tested is that lower-pitched voices are perceived as older and thus wiser and more experienced. Here the relationships between candidate voice pitch, candidate age, and electoral success are examined with two experiments. Study 1 tests whether voters discriminate on candidate age. The results show that male and female candidates in their 40s and 50s, the time in the lifecycle when voice pitch is at its lowest, are preferred over candidates in their 30s, 60s, and 70s. Study 2 shows that the preference for leaders with lower-pitched voices correlates with the perception that speakers with lower voices are stronger, more competent, and older, but the influence of perception of age on vote choice is the weakest of the three.

## Introduction

A growing literature in psychology and linguistics shows that voice pitch—the percept of “highness” or “lowness” of the human voice as determined by fundamental frequency (F_0_)—influences how speakers are perceived, that is, how they are thought of by listeners. Men with lower-pitched voices are perceived as more attractive [1], physically stronger [1], [2], and more dominant [3], [4]. Within this literature, a dominant individual is defined as one who “tells other people what to do, is respected, influential, and often a leader” [5]. For women, higher-pitched voices are perceived as more attractive [6], [7], [8], whereas lower-pitched female voices are perceived as more dominant [9], [10]. In both sexes, higher-pitched voices are associated with negative emotions such as panic, fear, and stress [11], [12], [13], [14], [15].

Importantly, human voice pitch also has been shown to influence the selection of leaders [4], [16], [17], [18], [19], [20]. By examining recordings of presidential debates between 1960 and 2000, Gregory and Gallagher [17] found that male candidates with lower voices performed better in public opinion surveys and won a higher percentage of the popular vote. Tigue et al. [4] presented experimental subjects with pairs of male voices that were manipulated digitally to vary in pitch. They found that men and women prefer to vote for the lower-pitched male voices. Klofstad et al. [18] and Anderson and Klofstad [16] replicated this finding with both male and female voices. Experiments by Laustsen et al. [20] show that the preference for leaders with lower-pitched voices is stronger among Republicans compared to Democrats. Klofstad [19] shows that 2012 United States House of Representatives candidates with lower voices won a larger vote share when facing male opponents, even after controlling for other influences on electoral outcomes such as campaign spending, district ideology, and incumbency.

Taken together, candidate voice pitch appears to influence voters both in the laboratory and in real life. But why do humans prefer leaders with lower-pitched voices? Tigue et al. [4] showed that this preference correlates with the perception that men with lower voices have greater physical prowess, and Klofstad et al. [18] show that men and women with lower voices are perceived as stronger. Individuals with lower voices have higher levels of testosterone [2], are physically stronger [1], [2], and are more aggressive [2], [21], [22], so there is a potential mechanism enabling voice pitch to be a reliable signal of physical prowess and strength. However, as modern political conflict is a competition between ideologies as opposed to brute force, it is unclear whether these correlates of voice pitch are functionally indicative of leadership ability in today’s world. More relevantly, Tigue et al. [4] show that the preference for leaders with lower-pitched voices correlates with the perception that men with lower voices have greater integrity, and Klofstad et al. [18] showed that men and women with lower voices are perceived as more competent. Integrity and competence are desirable leadership qualities, although the mechanism reliably linking voice pitch to these qualities is unclear. That is, there is no evidence to suggest how individuals with lower voices could be intrinsically more competent or have greater integrity.

An alternative hypothesis yet to be tested is that perception of age underlies the preference for leaders with lower-pitched voices. Voice pitch is a reliable signal of age because F_0_ decreases greatly from childhood to middle-age, and then increases to a lesser degree after middle-age, due to changes in hormonal profiles and concomitant effects on the anatomy and physiology of the vocal tract and larynx [23], [24], [25]. Further, a speaker’s age can be predicted accurately from the sound of his or her voice [24], [26], [27], [28], [29], [30], [31], [32], [33]. Because older individuals generally are perceived as wiser than younger individuals [34], and older speakers are perceived as wiser than younger speakers [35], the influence of voice pitch on voter preference may be driven, at least in part, by perception of age.

Here we examine the relationships between candidate voice pitch, candidate age, and electoral success with two experiments. Piliavin [36] and Sigelman and Sigelman [37] show that voters tend to prefer candidates of their own age. To the best of our knowledge, however, these are the only direct tests of whether candidate age matters to voters. As such, before testing whether perceptions of age underlay voters’ preference for candidates with lower voices, in Study 1 we first test whether voters discriminate on candidate age. We find that male and female candidates in their 40s and 50s, the time in the lifecycle when voice pitch is at its lowest, are preferred over candidates in their 30s, 60s, and 70s. Having established that candidate age can matter to voters, in Study 2 we test whether perceptions of candidate age account for voters’ preference for leaders with lower-pitched voices. We find that the preference for leaders with lower-pitched voices correlates with the perception that speakers with lower voices are stronger, more competent, and older, but the influence of perception of age is the weakest of the three.

## Study 1: Influence of Candidate Age on Vote Choice

### Participants

The experiment was administered online to 800 listeners (400 men and 400 women) by Qualtrics, an online portal for researchers to develop and administer survey questionnaires through the Internet. For this experiment Qualtrics partnered with Survey Sampling International (SSI) to recruit the participants. SSI maintains panels of subjects that are only used for research. Individuals voluntarily join a SSI panel by responding to an online SSI advertisement (e.g., a banner advertisement on a website). Participants received between $.50–.$75 USD in exchange for voluntary participation in this study. They were invited to participate in the study by email. To increase external validity Qualtrics drew participants evenly from three age groups: 18–33 (N = 265, 133 women and 132 men), 34–50: (N = 268, 134 women and 134 men), and 51–65 (N = 267 133 women and 134 men).

### Experimental design

Subjects were assigned randomly to vote in one of 90 different mock elections between two hypothetical candidates. The mock elections included every permutation of men and women aged 30, 40, 50, 60, and 70 years of age. For example, a participant could have been asked, “If they were running against each other in an election, who would you vote for, a 30 year old man, or a 30 year old woman?” The only information presented to the subject was the age and sex of the candidates. The order of the candidates on the mock ballot was randomized. Elections in which the candidates were the same age and sex (e.g. a 30 year old woman versus a 30 year old woman) were not included in the experiment.

### Statistical analysis

Analyses were conducted in SPSS (v. 19). Analysis of variance (ANOVA) is used to assess variation in the outcomes of the mock elections. The candidate is used as the unit of analysis to assess the influence of candidate age and sex on electoral outcomes. The voter is used as the unit of analysis to assess the interaction effect of voter age and candidate age on electoral outcomes.

### Ethics Statement

Prior approval to conduct all elements of this experiment was granted by the Duke University (Durham, North Carolina, USA) Institutional Review Board on August 30, 2013 (Protocol #B0901). Survey Sampling International (SSI), the provider of the research subjects, complies fully with European Society for Opinion and Marketing Research (ESOMAR) standards for protecting individuals' privacy and information. Individuals voluntarily join a SSI panel. All communications between SSI and panel members explain why the member has been selected to participate in a study, what he or she should expect from membership in the panel, and offer multiple methods to opt-out of participating. Study participants provided written consent to participate by voluntarily clicking a link to the survey in the email invitation. Participants were free to stop participating at any time by closing their web browser program. Participation in the study was confidential. Identifying information, such as names or addresses, was not collected during the experiment.

### Results

**Fig 1:**
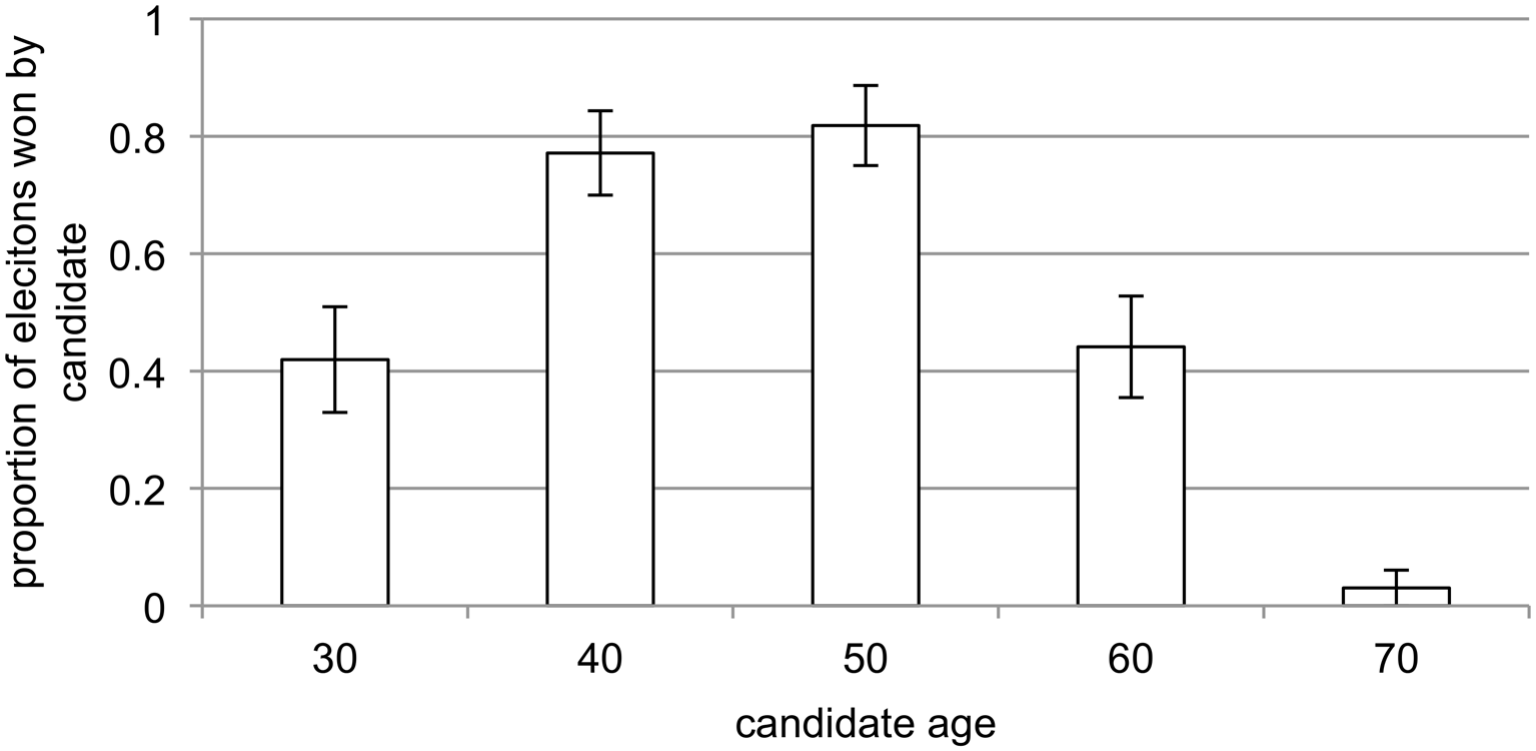
Proportion of elections won (+/- SE) as a function of candidate age.

**Fig 2:**
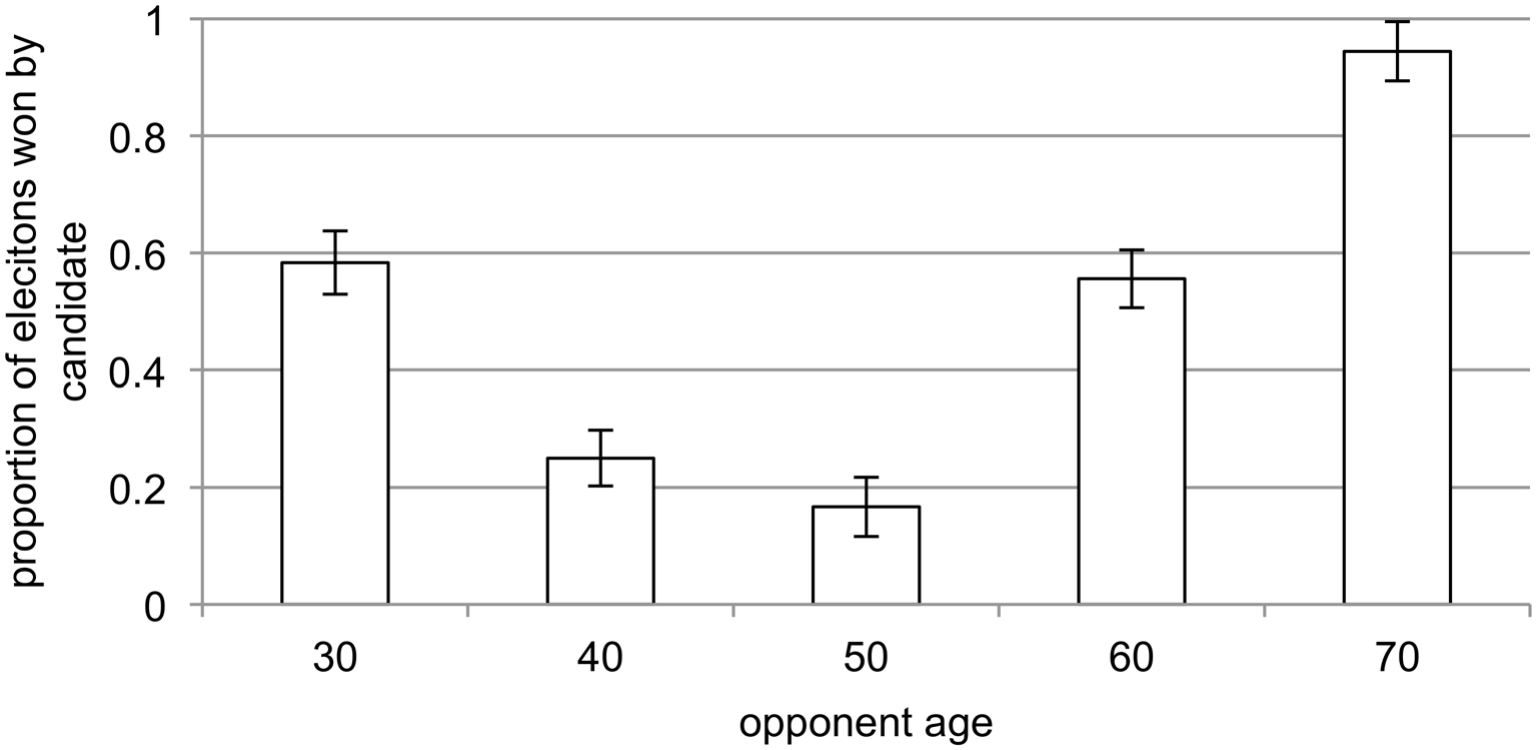
Proportion of elections won (+/- SE) as a function of opponent age.

Using the candidate as the unit of analysis, a three-way ANOVA of whether the candidate won with candidate age, opponent age, and a categorical variable indicating the sex of the candidate and the opponent (i.e. male vs. male, male vs. female, female vs. male, or female vs. female), shows a significant effect for candidate age (F_4,76_ = 25.71, p < .001, partial η^2^ = .58). Candidates in their 40s and 50s, the time in the lifecycle when voice pitch is the lowest [23], [24], [25], were the most successful (Fig 1). There was also a significant effect for opponent age (F_4,76_ = 25.92, p < .001, partial η^2^ = .58). Candidates were the most successful against opponents aged 30, 60, and 70 (Fig 2). The interaction of candidate age and opponent age was also statistically significant (F_16,76_ = 1.83, p = .04, partial η^2^ = .28). Candidates in their 30s were the least successful against opponents in their 40s and 50s, candidates in their 40s and 50s won at least 50% of the time regardless of the age of the opponent, candidates in their 60s were the least successful against opponents in their 40s and 50s, and candidates in their 70s only won election if facing opponents who were also in their 70s (Table 1).

**Table 1:** Proportion of elections won by candidate age and opponent age.

Candidate Age	Opponent Age				
	30	40	50	60	70
30	.50 (.14)	.13 (.10)	.00 (.12)	.50 (.12)	1.00 (.11)
40	.88 (.10)	.50 (.14)	.50 (.11)	.75 (.10)	1.00 (.10)
50	1.00 (.12)	.50 (.11)	.50 (.14)	1.00 (.10)	1.00 (.10)
60	.50 (.12)	.25 (.10)	.00 (.10	.50 (.14)	1.00 (.10)
70	.00 (.12)	.00 (.10)	.00 (.10)	.00 (.10)	.50 (.20)

Cell values are based on estimated ANOVA estimates, standard errors in parentheses.

The only other statistically significant factor in the ANOVA model was the three-way interaction between candidate age, opponent age, and the sex of the candidates (F_38,76_ = 2.14, p = .003, partial η^2^ = .52). The top half of Table 2 shows fewer differences in the effect of male and female candidates’ ages in same-sex races compared to the differences seen for opposite sex races in the bottom portion of Table 2. That is, the interaction effect of candidate age and opponent age described above appears to be mediated by the sex of the candidate and opponent, and especially so when men and women run against each other. For men, 50 year-old candidates were the most successful, regardless of the sex of their opponent. In contrast, while 50 year-old female candidates were the most successful against female opponents, 40 year-old women were the most successful against male opponents. The results also show that 70 year-old female candidates never won office against a male opponent, regardless of his age.

**Table 2:** Proportion of elections won by candidate age, opponent age, and sex of candidates.

Candidates of Same Sex					
male candidate age	male opponent age				
	30	40	50	60	70
30	—	.00 (.20)	.00 (.20)	.00 (.28)	1.00 (.28)
40	1.00 (.20)	—	.50 (.20)	.50 (.20)	1.00 (.20)
50	1.00 (.20)	.50 (.20)	—	1.00 (.20)	1.00 (.20)
60	1.00 (.28)	.50 (.20)	.00 (.20)	—	1.00 (.20)
70	.00 (.28)	.00 (.20)	.00 (.20)	.00 (.20)	—
female candidate age	female opponent age				
	30	40	50	60	70
30	—	.00 (.20)	.00 (.20)	.00 (.20)	1.00 (.20)
40	1.00 (.20)	—	.00 (.28)	1.00 (.20)	1.00 (.20)
50	1.00 (.20)	1.00 (.28)	—	1.00 (.20)	1.00 (.20)
60	1.00 (.20)	.00 (.20)	.00 (.20)	—	1.00 (.20)
70	.00 (.20)	.00 (.20)	.00 (.20)	.00 (.20)	—
Candidates of Opposite Sexes					
male candidate age	female opponent age				
	30	40	50	60	70
30	1.00 (.20)	.00 (.20)	.00 (.28)	1.00 (.20)	1.00 (.20)
40	.50 (.20)	.00 (.20)	.50 (.20)	1.00 (.20)	1.00 (.20)
50	1.00 (.28)	.00 (.20)	1.00 (.20)	1.00 (.20)	1.00 (.20)
60	.00 (.28)	.50 (.20)	.00 (.20)	.50 (.20)	1.00 (.20)
70	.00 (.28)	.00 (.16)	.00 (.20)	.00 (.20)	1.00 (.20)
female candidate age	male opponent age				
	30	40	50	60	70
30	.00 (.20)	.50 (.20)	.00 (.28)	1.00 (.28)	1.00 (.20)
40	1.00 (.20)	1.00 (.20)	1.00 (.20)	.50 (.20)	1.00 (.20)
50	1.00 (.28)	.50 (.20)	.00 (.20)	1.00 (.20)	1.00 (.20)
60	.00 (.20)	.00 (.20)	.00 (.20)	.50 (.20)	1.00 (.20)
70	.00 (.20)	.00 (.20)	.00 (.20)	.00 (.20)	.00 (.20)

Cell values are based on estimated ANOVA estimates, standard errors in parentheses.

**Fig 3:**
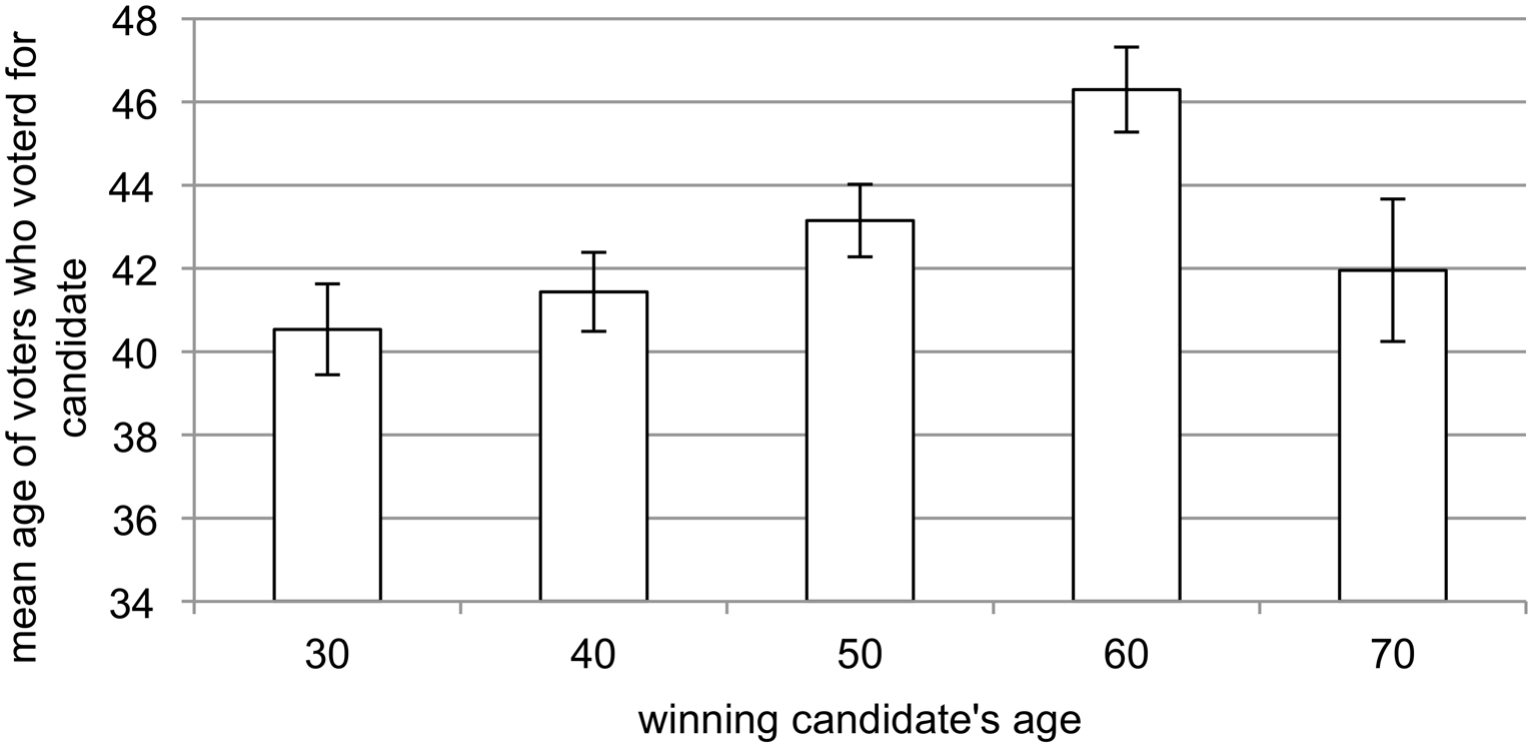
Mean age of voters (+/- SE) who voted for the candidate as a function of candidate age.

Treating the voter as the unit of analysis allows for an assessment of the relationship between a voter’s age and his or her preferences for candidates of different ages. An ANOVA of age of the voter with the age and sex of the candidate the voter selected, and the age and sex of the candidate they rejected, as the factors shows a significant effect for the age of the candidate the voter selected (F_4,711_ = 5.38, p < .001, partial η^2^ = .03) and the age of the candidate the voter rejected (F_4,711_ = 3.23, p = .012, partial η^2^ = .02). More specifically, in line with previous studies [36], [37], there was a positive relationship between candidate age and voter age (Fig 3), although the mean age of voters who supported 70 year-old candidates is younger than that of voters who supported 60 year-old candidates. None of the other factors in the ANOVA model were statistically significant.

## Study 2: Influence of Perceived Strength, Competence, and Age on Preference for Candidates with Lower Voices

### Participants

The experiment was administered online to 803 subjects (400 men and 403 women) by Qualtrics. For this experiment Qualtrics partnered with Sample Strategies to recruit research subjects. Previous studies on voice pitch and the selection of leaders used samples of college undergraduates [4], [16], [18]. In contrast, Sample Strategies recruited a general population sample of participants from across the United States. Participants ranged in age from 18 to 78 years ($\bar{x}$ = 49 years, SE = .49 years), and received a cash incentive after participating ($1.25 USD). They were invited to participate in studies by email and through messages on their Sample Strategies account homepage.

### Experimental design

Five men and 5 women were recorded speaking the sentence, “I urge you to vote for me this November,” a politically relevant yet partisan-neutral statement. The women ranged in age from 21 to 38 years ($\bar{x}$ = 26 years, SE = 3), and the men 20 to 41 years ($\bar{x}$ = 28 years, SE = 4). Voices were recorded as.wav files in an Acoustic Systems soundproof room using a Shure SM57 microphone and a Marantz PMD660 solid-state recorder. Each audio file was inspected aurally and visually in Audacity (v. 2.0.1; audacity.sourceforge.net) to ensure that they were free from speech errors and non-speech noise. Engineering Design’s Signal acoustics analysis program (v. 4.02.04; www.engdes.com) was used to normalize the amplitude (i.e. “loudness”) of the recordings. The pitch of female and male voices range from 189 to 207 Hz ($\bar{x}$ = 199 Hz, SE = 3 Hz) and 91 to 116 Hz ($\bar{x}$ = 107 Hz, SE = 4 Hz), respectively. Praat (v. 5.1.43) [38] was used to measure pitch.

Following previous studies [39], each recording was altered +/-.5 equivalent rectangular bandwidths (ERB) with Praat, which uses the Pitch Synchronous Overlap Add Method (PSOLA) algorithm to alter F_0_ [38]. The relationship between absolute and perceived pitch in humans is logarithmic. Manipulation by ERB accounts for this fact and produces a constant perceivable gap between the higher- and lower-pitched sound files regardless of the F_0_ of the original recording. The pitch of the higher- and lower-pitched female sound files range from 214 to 233 Hz ($\bar{x}$ = 224 Hz, SE = 3 Hz) and 170 to 190 Hz ($\bar{x}$ = 181 Hz, SE = 3 Hz) respectively. The pitch of the higher- and lower-pitched male sound files range from 110 to 136 Hz ($\bar{x}$ = 127 Hz, SE = 4 Hz) and 81 to 98 Hz ($\bar{x}$ = 91 Hz, SE = 3 Hz) respectively.

A previous study verified that voters can perceive which voice of each pair used in this experiment is higher in pitch [18]. The magnitude of this perceived shift gap is roughly equivalent to the musical interval difference between open string G and D notes on a guitar. On a piano, this interval is roughly equivalent to the difference between C4 (“Middle C”) and A3.

Participants were assigned randomly to listen to either the pairs of male (N = 201 male and 202 female voters) or female (N = 199 male and 201 female voters) voices. Before participating, voters completed a sound check task to insure that they could hear audio played by the online survey instrument. Voters chose whether to use computer speakers (N = 676) or headphones (N = 127). Online voice pitch experiments produce results that are comparable to laboratory experiments [40]. After listening to a pair of voices, voters were asked which person of each pair was stronger, more competent, older, and for whom they would vote. Participants rated multiple pairs of voices to reduce biases due to pseudoreplication, whereby the idiosyncratic characteristics of any one pair of voices might influence the results of the experiment [41], [42]. A specific type of leadership role was not referred to in the vote question to remove this potentially confounding factor from the experiment. These questions were asked individually. As such, each participant made twenty forced choices (four questions asked of five voice pairs). The order of the pairs of voices, whether the higher-or lower-pitched version of each voice was presented first, and the order of the questions asked about the voices were randomized.

### Statistical analysis

The participant is the unit of analysis. Analyses were conducted using SPSS (v. 19). Choices were coded 1 if the participant selected the lower voice and 0 if the higher voice was selected. The average of these choices for each voter yields a summary preference ratio ranging from 0 to 1, whereby higher values indicate a stronger preference for lower-pitched voices. A preference ratio of .5 indicates that the voter had no preference for either the higher or lower voices. One-sample t-tests, ANOVA, and multivariate linear regression were used to assess voters’ preferences. All tests are two-tailed. Because participants were not obliged to answer every question in the survey, only participants that answered every question about the characteristic of the voice were used (strength: N = 786; competence: N = 786; older: N = 790; vote for: N = 788). Sample sizes vary because cases with missing data were omitted from each analysis using listwise deletion.

### Ethics Statement

Prior approval to conduct this experiment was granted by Duke University (Durham, North Carolina, USA) Institutional Review Board on October 7, 2013 (Protocol B0948). Sample Strategies, the provider of the research subjects, complies fully with European Society for Opinion and Marketing Research (ESOMAR) standards for protecting individuals' privacy and information. Individuals voluntarily join a Sample Strategies panel. All communications between Sample Strategies and panel members explain why the member has been selected to participate in a study, what he or she should expect from membership in the panel, and offer multiple methods to opt-out of participating. Study participants provided written consent to participate by voluntarily clicking a link to the survey in the email invitation. Participants were free to stop participating at any time by closing their web browser program. Participation in the study was confidential. Identifying information, such as names or addresses, was not collected during the experiment.

### Results

Table 3 reports the proportion of times the lower-pitched voice was selected as stronger, more competent, older, and voted for. The data show that male and female participants found the lower-pitched male and female voices to be stronger, more competent, older, and more electable.

**Table 3:** Proportion of lower-pitched voices judged as competent, strong and trustworthy.

	Male candidates		Female candidates	
	male voters	female voters	male voters	female voters
Strong	.72***	.72***	.74***	.78***
Competent	.62***	.61***	.71***	.76***
Older	.78***	.77***	.83***	.84***
Voted for	.61***	.60***	.67***	.76***

A value of 0.50 represents no discernible preference for either higher- or lower-pitched voices.

***p ≤ 0.001 (two-tailed one-sample t-tests with comparison value of .50).

ANOVAs were conducted on the participants’ preferences for strength, competence, age, and voting. Each analysis included participant sex, speaker sex, and the interaction of the two as the factors. Type of playback device (headphones or computer speakers) was included as a covariate. The results show that the preference for lower-pitched voices is significantly stronger when judging female voices (strength: F_1,768_ = 4.77, p = .029, partial η^2^ = .01; competence: F_1,786_ = 40.08, p < .001, partial η^2^ = .05; older: F_1,790_ = 16.00, p < .001, partial η^2^ = .02; vote for: F_1,788_ = 30.78, p < .001, partial η^2^ = .04). There was no effect of participant sex when judging strength (F_1,768_ = 1.47, p = .23, partial η^2^ = .002), competence (F_1,786_ = 1.22, p = .27, partial η^2^ = .002), or age (F_1,790_ = .21, p = .65, partial η^2^ < .001). Likewise, there were no interaction effects between speaker sex and participant sex when judging strength (F_1,768_ = 1.21, p = .27, partial η^2^ = .002), competence (F_1,786_ = 2.79, p = .10, partial η^2^ = .004), or age (F_1,790_ = .62, p = .43, partial η^2^ = .001). In contrast, there is an effect for participant sex when voting (F_1,788_ = 4.87, p = .028, partial η^2^ = .01), whereby the preference for leaders with lower voices is strongest among female participants. There is also an interaction effect between speaker sex and participant sex when voting (F_1,788_ = 6.57, p = .01, partial η^2^ = .01), whereby the preference for leaders with lower voices is the strongest when women are judging the voices of other women.

Bivariate correlations show that the preference to vote candidates with for lower-pitched voices correlates with the perception that they are stronger, more competent, and older (Table 4). Multivariate linear regression was used to further assess the relationship between participants’ vote choices and their perceptions of strength, competence, and age in the voices (Table 5). Type of playback device (headphones or computer speakers) was included in the analyses as a covariate. This analysis allows for a comparison of the relative influence that perceptions of strength, competence, and age have on vote choice. The first column of coefficients in Table 5 indicates that leaders with lower voices are preferred because they are perceived as stronger, more competent, and older. The effect of perceptions of age, however, is less than half of that of perceptions of a candidate’s competence and strength.

**Table 4:** Correlations between vote choice and perceptions of strength, competence, and age.

	Strong	Competent	Older	Voted for
Strong	—			
Competent	.43***	—		
Older	.35***	.33***	—	
Voted for	.44***	.45***	.30***	—

Cell values are Pearson correlation coefficients.

***p ≤ 0.001 (two-tailed).

**Table 5:** Multivariate analysis of vote choice preference ratio.

	All observations	Male candidates		Female candidates	
		male voters	female voters	male voters	female voters
Strength preference ratio (weak-strong)	.30*** (.04)	.32*** (.08)	.33*** (.07)	.36*** (.08)	.19** (.07)
Competence preference ratio (incompetent-competent)	.31*** (.04)	.34*** (.08)	.23** (.07)	.31*** (.08)	.25*** (.07)
Age preference ratio (young-old)	.13*** (.04)	.16* (.07)	.06 (.08)	.09 (.09)	.16^ (.09)
Playback device (headphones)	-.02 (.02)	-.01 (.04)	-.04 (.05)	.04 (.05)	-.06 (.04)
Constant	.12*** (.04)	.04 (.07)	.18** (.07)	.10 (.08)	.29*** (.08)
Adjusted R^2^	.29	.32	.22	.28	.20
F	77.35***	23.05***	14.62***	18.94***	13.21***
N	764	188	191	187	195

Cell values are linear regression coefficients, standard errors in parentheses. Variables were entered into the model simultaneously. Observations with missing data are excluded from the analysis by listwise deletion.

^p ≤ .10;

*p ≤ .05;

**p ≤ .01;

***p ≤ .001

Looking across the first row of coefficients in Table 5, the data show that male and female voters prefer male and female candidates with lower voices because they are perceived as stronger. Perceptions of strength, however, appear to matter less when women are judging other women. The second row of coefficients in Table 5 indicates then male and female voters prefer male and female candidates with lower voices because they are perceived as more competent. Perceptions of competence, however, appear to matter less when women are judging male and female candidates. The third row of Table 5 indicates that perceptions of candidate age only significantly affect the votes of men voting for men. A trend (p = .076) suggests the same for women judging women.

## Discussion

Here we examined whether perception of age underlies the preference voters show for leaders having lower-pitched voices. We show in Study 1 that, when asked to discriminate on candidate age in the absence of any other information, voters prefer leaders in their 40s and 50s. This result is in line with actual electoral outcomes. For example, the mean age of current members of the United States House of Representatives is 57.0 years, and 49.3 years among those who were elected for the first time in 2012 [43]. These findings are consistent with the idea that voters prefer leaders who are neither too young and inexperienced, nor too old and less capable of active leadership. The 40s and 50s are also the point in the human lifecycle in both men and women when voice pitch is at its lowest [23], [24], [25], suggesting that a preference for leaders with lower voices could reflect a preference for leaders who have age and experience but are not too aged to lead effectively.

Study 1 also revealed variation in voters’ preference based on an interaction between the age and sex of the candidates. While male and female candidates performed equally well overall, male candidates in their 50s were the most successful regardless of the sex of their opponent, and female candidates in their 40s were the most successful when facing male opponents. Moreover, while candidates in their 70s were unlikely to win, female candidates in their 70s never won. Taken together, these results suggest a bias in favor of younger women in elected office, especially when they face male opponents. Given the negative relationship between perceived age and perceived attractiveness [44], and the bias in favor of attractive female candidates [45], a preference for younger female candidates could reflect a preference for physically attractive female leaders.

In line with previous studies [36], [37], we also found evidence in Study 1 of positive assortment by age between voters and candidates. This result is consistent with research showing that voters tend to prefer leaders who match their demographic characteristics under the assumption that they may be more likely to also share their political interests [46]. For example, a younger leader may be more attentive to issues germane to younger voters such as student loan rates and access to education, while an older leader may be more attentive to issues germane to older voters such as retirement benefits and healthcare.

Also in line with previous studies [4], [16], [17], [18], Study 2 confirmed that voters prefer leaders with lower voices. Importantly, here we also found that this bias is strongest in the case of female candidates. Because voice pitch is highly sexually dimorphic, on average twice as high in women as compared to men [25], this result suggests that voice pitch may be an impediment to women gaining positions of leadership. To be clear, we do not suggest that voice pitch on its own could explain the unequal representation of the sexes in governments across the globe. Our data do show, however, that voice pitch—an anatomically and physiologically determined characteristic—may enhance the advantage of men over women in seeking positions of leadership. As a corollary, the bias in favor of leaders with lower-pitched voices benefits female candidates who happen to be physically larger [2], between the ages of 40 and 60 [23], [24], [25], and who have higher levels of testosterone [2], all of which are correlates of lower pitched voices.

Most importantly, the results of Study 2 do not support the idea that the established preference for leaders with lower voices might be best explained by the correlation of voice pitch with age. Both men and women with lower voices were deemed by subjects to be stronger, more competent, and older. However, perceptions of candidate strength and competence explain more of the variation in voters’ vote choices than perceptions of candidate age.

If older individuals are perceived to be wiser [34], [35] and thus more capable of leadership, why is the perception of age a weaker influence on our preference for leaders with lower voices? This could be because perceptions of age are not uniformly positive. For example, while age is correlated positively with perceptions of wisdom, it is correlated negatively with perceptions of attractiveness, power, and health [34], [35]. These mixed perceptions of age are reflected in the results from Study 1. Voters preferred leaders who are old enough to have the experience to lead, but not too old and thus incapable of active leadership.

Study 2 also showed that the influence of perception of age on voters’ preference for candidates with lower voices only appears to matter when voters assess candidates of their own sex. While the magnitude of this effect is the same for male and female voters, it is more certain statistically when men judge men than when women judge women. Why this apparent within-sex enhancement of assessment occurs is unclear, although we might speculate that individuals may be more attuned to subtle voice differences of their same sex.

Taken together with prior work, our results indicate that even though voters can discriminate on age based on voice pitch and prefer candidates at ages when voice pitch is lowest, perception of age plays a lesser role in determining the preference for candidates with lower pitched voices. Instead, perceptions of strength and competence appear to explain why voters prefer leaders with lower voices. This result may be surprising, as it is unlikely that individuals with lower pitched voices are inherently more competent leaders, and physical strength is also unlikely to be germane to modern-day political leadership. Rather, the preference for leaders with lower voices more likely reflects correlates between voice quality and leadership capability that were relevant at some earlier time in human evolutionary or cultural history. This said, as individuals with lower voices have higher levels of testosterone [2] and are more aggressive physically and socially [2], [21], [22], using voice pitch as a signal of leadership might lead to the selection of leaders who are more aggressive in pursuing the interests of their constituents. As such, the question remains as to whether individuals with lower voices are actually more capable leaders in the modern world. Future studies can address this by correlating the qualities of people’s voices with objective measures of their leadership abilities.

In this same vein, future studies should build upon the results presented here by using more sophisticated research designs that mimic more closely the conditions of real elections. For example, Study 2 should be replicated pairing male and female candidates against each other to see how the influence of voice pitch interacts with the influence of candidate sex. Also, knowing that physical appearance influences electability [47], [48], [49], [50], [51], candidate voices and faces could be presented in tandem to compare the effects of visual and vocal stimuli. Knowing that the perceptions of voice pitch can be influenced by the content of the utterances presented to study participants [39], future studies could also alter the political messages in the stimuli. For example, as leaders with more masculine faces are preferred in time of war [49], [52], it could be that the preference for leaders with lower (i.e. more masculine) voices is stronger when the candidate speaks about foreign rather than domestic policy. While it is clear that candidate voice pitch can influences voters more study is needed to understand the conditions under which this vocal signal is influential, and what consequences this voter bias has for how societies are governed.
